# Treatment allocation in ophthalmological randomised-control trials (TAO-RCT): A cross-sectional meta-research study

**DOI:** 10.1038/s41433-025-03922-y

**Published:** 2025-07-17

**Authors:** Arun James Thirunavukarasu, Santosh Guru, Henry Rocha, Chandan Sekhon

**Affiliations:** 1 Nuffield Department of Clinical Neurosciences, Medical Sciences Division, University of Oxford, Oxford, UK; 2 Oxford University Hospitals NHS Foundation Trust, Oxford, UK; 3 University of Cambridge School of Clinical Medicine, University of Cambridge, Cambridge, UK

**Keywords:** Medical research, Education, Eye diseases

## Abstract

**Purpose:**

Ophthalmological randomised-control trials (RCTs) are complicated by inter-eye correlation, contralateral crossover effects, and heterogeneity in treatment allocation patterns. This study examined the prevalence of uncontrolled treatment allocation and its impact on outcomes in ophthalmological RCTs.

**Methods:**

All ophthalmological RCTs published in 2022 were analysed in a cross-sectional study that adhered to a preregistered protocol (CRD42023474661). Eligible trials were assessed for treatment allocation patterns and clarity of reporting using a simple nomenclature system. Associations between uncontrolled allocation and study characteristics, reporting clarity, funding, and trial outcomes were examined.

**Results:**

From 359 RCTs, 42 distinct allocation patterns were identified, with 306 trials (85.2%) using controlled treatment allocation. Uncontrolled treatment allocation was associated with unclear reporting (χ^2^ = 44.7, *p* < 0.001) and two-eye allocation patterns (Fisher’s exact test, *p* < 0.001). The distribution of *p*-values was similar between controlled and uncontrolled trials (*t* = −0.603, *p* = 0.547), suggesting no increased likelihood of statistical significance. Uncontrolled allocation was more frequent in non-English-speaking countries (χ^2^ = 4.681, *p* = 0.030) and studies of surgical interventions (χ^2^ = 4.287, *p* = 0.038).

**Conclusion:**

Uncontrolled treatment allocation is prevalent in ophthalmological RCTs and is associated with unclear reporting, two-eye study designs, non-English-speaking settings, and surgical interventions. While no signs of deliberate misuse are evident, these patterns may compromise the validity of trial analysis. Transparent reporting and careful consideration of treatment allocation should be prioritised in trial design, protocol registries, and appraisal frameworks. A standardised nomenclature system could improve clarity and reproducibility.

## Introduction

In ophthalmology, randomised-control trials (RCTs) are complicated by statistical quirks resulting from the fact that most patients have two eyes [1]. Investigators have a range of options to design and analyse trials. For instance, participants’ eyes may be treated differently to leverage a paired control; participants (rather than eyes) may be randomised to unilateral or bilateral treatment with no inter-eye comparison; or investigators may treat and/or measure just one eye from each participant to simplify statistical analysis [2]. Interventions may themselves be unilateral or bilateral, as can ophthalmological pathology. Similarly, trial endpoints can involve one or both eyes, and many clinical endpoints—such as higher order visual task performance—depend on bilateral vision [3].

The simplest randomised-control trials compare outcomes following an index intervention against a control arm which is treated according to current best clinical practice (with a placebo if necessary to support blinding) [4]. Assigning one eye per individual to each arm is attractive as it generates paired controls with few or no confounding factors. However, the contralateral eyes and vision of each individual often correlate in terms of disease and function, compromising many statistical tests which depend on independence of measurements for their validity [1]. Moreover, systemic treatments cannot be selectively directed towards a single eye, and topical or local treatments can have contralateral crossover which mask observed clinical effects [5]. Further complication arises where there are more than two interventions being trialled, as intercorrelation between participants’ eyes may affect some but not all statistical comparisons. Finally, randomisation of eyes rather than participants can create imbalance in treatment exposure, with some participants contributing more data points than others.

There is no single correct treatment allocation pattern in ophthalmological trials. The optimal treatment allocation pattern depends on characteristics of the diseases, interventions, and outcomes of interest [6]. However, treatment allocation patterns may be categorised in broad terms as either *controlled* or *uncontrolled*. Specifically, with controlled treatment allocation patterns, the methodological and statistical relationship between interventions is consistent; such that observed differences can be more reliably attributed to the intervention. Conversely, uncontrolled treatment allocation patterns occur where eyes are irregularly assigned to study arms, such as through inconsistent unilateral and bilateral treatment or variable overlap of treatment arms within individuals.

Uncontrolled treatment allocation results in statistical noise associated with confounding factors, as a subset of the eyes being compared are exposed to variable crossover effects and performance bias. This can lead to spurious results, leading to mistreatment of patients which may continue for many years before being corrected [7]. Despite these potential issues, treatment allocation patterns are not emphasised in trial design, and often not described in reports of clinical trals [2, 6]. In addition, statistical analysis is often inappropriate with respect to treatment allocation, compromising a large proportion of results [2, 8].

Here, a cross-sectional study was undertaken to explore the characteristics of treatment allocation in ophthalmological randomised-control trials (TAO-RCT). By characterising and quantifying the frequency of uncontrolled treatment allocation, we aimed to prompt investigators to consider this novel aspect of trial design to optimise the informativeness of experimental results. We further aimed to investigate if there was any evidence of treatment allocation being purposefully abused, such as to increase the likelihood of positive results occurring due to statistical noise. We made use of a simple and concise system of nomenclature to allow investigators, appraisers, and reviewers to easily describe the treatment allocation of eye-related randomised-control trials.

## Methods

### Searching and screening

A cross-sectional study was undertaken of ophthalmological randomised-control trials reported within a 12-month period [9]. The search and screening process adhered to a protocol preregistered on PROSPERO (CRD42023474661). A comprehensive search strategy was designed to capture ophthalmological randomised-control trials reported in 2022 (Supplementary Material 1). Abstract and full text screening was undertaken by two blinded researchers, with a third independent researcher casting a deciding vote to resolve disagreement. The following inclusion criteria were used for screening:

1. Published between 1 January 2022 and 31 December 2022
2. Written in the English language
3. Human participants featured
4. Ophthalmological intervention featured
5. Prospective study design
6. Randomised allocation of intervention and control arm treatments

### Data collection

Data collection for each paper was undertaken by a single researcher (SG, HR, or CS), with a second researcher (AJT) subsequently verifying each entry. A spreadsheet was used to collect the following data from each study: country of study, funding source (academic, governmental, or industrial), condition of interest, intervention/s tested, comparator/s, subspecialty and whether the intervention was medical or surgical, allocation pattern (as described below), clarity of treatment allocation pattern (explicit, implicit, or unclear), primary outcome/s, and *p* values of tests comparing primary outcomes between intervention.

We developed a simple and concise nomenclature system for describing treatment allocation patterns: descending letters from ‘A’ to describe treatments (including any placebo), ‘X’ to denote no treatment, ‘+’ between letters corresponding to contralateral eyes within an archetypical individual, and ‘&’ to separate archetypical individuals. For instance, ‘A + A & B + B’ would indicate that individuals were randomised to receive treatment ‘A’ or ‘B’ in both eyes; ‘A + X & B + X’ would indicate that individuals were randomised to receive treatment ‘A’ or ‘B’ in one eye, with the contralateral eye untreated. In addition, treatment allocation patterns were described in accordance with previous studies of one-eye or two-eye allocation of eyes in RCTs; to facilitate comparisons across different time points [2, 8].

Controlled and uncontrolled treatment allocation patterns were differentiated on the basis of whether they exhibited a consistent relationship between interventions and participants. For instance, simple controlled allocation patterns included ‘A + B’ and ‘A + A & B + B’; whereas exemplar uncontrolled allocation patterns included ‘A + A & B + B & A + B’. This is because variable crossover effects of interventions A and B could influence the effectiveness and therefore the results of statistical comparisons between A and B.

### Statistical analyses and data visualisation

The frequency of different treatment allocation patterns was first computed and compared with previous reviews of ophthalmological study design. Chi-squared statistics or Fisher’s exact tests (where frequency lower than 5 was present in at least one cell of a two-way table) were used to describe the association between uncontrolled treatment allocation and six potential risk factors: clarity of reporting, one-eye versus two-eye allocation pattern, country and region of origin, ophthalmological subspecialty, and medical versus surgical intervention. *p*-values for primary endpoints were extracted from each trial and analysed to evaluate whether statistical significance was more likely in trials featuring uncontrolled treatment allocation using a *t*-test. Throughout the analysis, a *p*-value of less than 0.05 was accepted as statistically significant, but all analyses were also considered with respect to observed effect sizes. Prospective power analysis was not undertaken because sample size was determined by the number of eligible studies returned in the initial literature search. Analysis was conducted in R (version 4.1.2; R Foundation for Statistical Computing) and figures were produced in Affinity Designer (version 1.10.6; Pantone LLC).

## Results

### Study characteristics

**Fig. 1:**
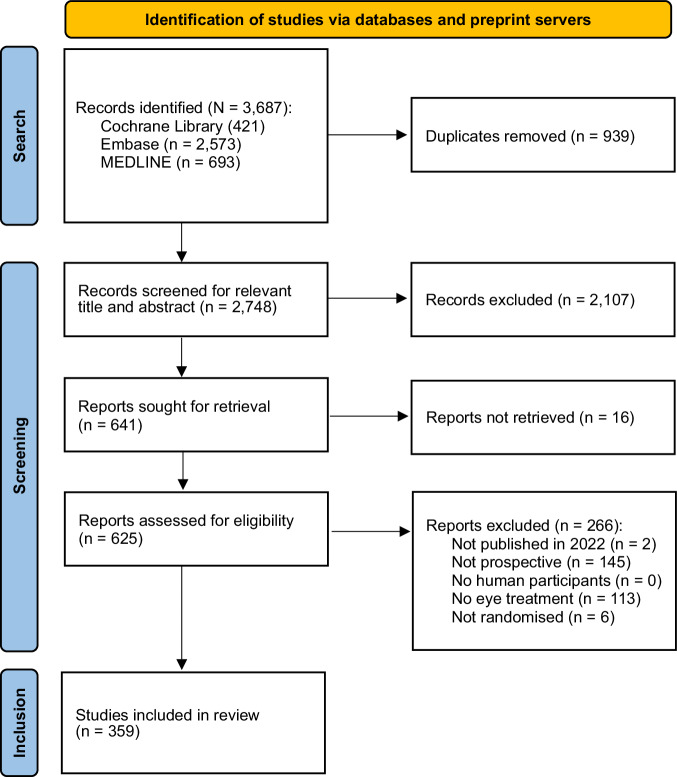
PRISMA flow chart depicting the search and screening process of identifying eligible records for the cross-sectional study. 359 ophthalmological randomised-control trials were ultimately identified and included in subsequent analyses. 16 records could not be retrieved for full text screening after contacting the corresponding authors.

Of 3,687 studies retrieved by the search strategy, 359 were ultimately included. The search and screening process is summarised in Fig. 1. A summary of the studies’ characteristics is provided in Table 1; with details for each included study available in Table S1. China and the United States were the most frequent countries of study by a large margin. Most studies (58.2%) declared some form of funding support, most frequently from industry. Medical interventions (n = 262) were much more frequent than surgical interventions (n = 97). The most frequent subspecialty of study was cornea, followed by retina. The number of authors per paper ranged from one to 124, but the median number of authors was seven.

**Table 1:** A summary of study characteristics for all 359 ophthalmological randomised-control trials included in the cross-sectional study.

**Study characteristic**												
Medical versus surgical intervention	Medical	Surgical										
	262	97										
Subspecialty	Cataract	Cornea	Glaucoma	Neurology	Oculoplastics	Paediatrics	Retina	Strabismus	Uveitis			
	47	116	37	4	22	48	76	7	2			
Location	Argentina	Australia	Austria	Belgium	Brazil	Canada	China	Croatia	Czechia	Denmark	Egypt	France
	1	6	1	1	6	5	66	2	2	3	26	3
	Germany	Greece	Hong Kong	India	Indonesia	Iran	Italy	Japan	Mexico	Nepal	Netherlands	Norway
	7	4	4	36	1	16	9	8	1	2	4	2
	Pakistan	Poland	Portugal	Singapore	South Korea	Spain	Sweden	Syria	Taiwan	Thailand	Turkey	United Kingdom
	8	1	1	4	10	7	1	1	1	2	11	11
	United States	Multinational										
	52	33										
Funding	Academic	Governmental	Industrial	Combination	None							
	43	34	89	43	150							
Clarity of reporting of treatment allocation pattern	Explicit	Implicit	Unclear									
	160	132	67									
Treatment allocation pattern	Controlled	Uncontrolled										
	306	53										

### Uncontrolled treatment allocation is associated with two-eye design and unclear reporting

**Fig. 2:**
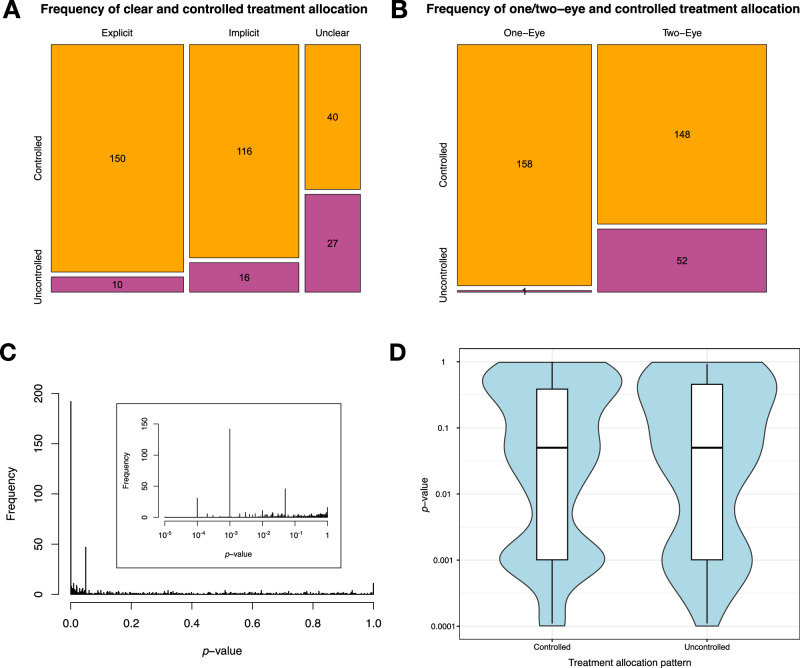
Distribution of treatment allocation and *p*-values of ophthalmological randomised-control trials. Mosaic plots depicting the distribution of controlled (yellow) and uncontrolled (magenta) treatment allocation patterns in ophthalmological randomised control trials, stratified by clarity of reporting of allocation pattern in panel **A** and by one-eye and two-eye allocation (per participant) in panel **B**. Uncontrolled allocation occurred in 52 (14.5%) trials in 2022. Uncontrolled allocation was more frequent in trials where reporting is implicit or unclear; and in trials exhibiting allocation of treatment to two eyes per participant. **C** Distribution of *p*-values for co-primary endpoints in ophthalmological randomised-control trials included in the study. The inserted histogram in represents the same data but with *p*-values plotted on a logarithmic x-axis. Peaks corresponding to common accepted levels of statistical significance (0.05, 0.01, 0.001) are suggestive or *p*-hacking or reporting bias. **D** *p*-values for co-primary endpoints stratified by controlled and uncontrolled treatment allocation patterns. Similar distributions of *p*-values in controlled and uncontrolled trials evidence against an increased likelihood of statistically significant results in uncontrolled trials.

In 359 RCT reports, 42 distinct treatment allocation patterns were identified (Table 2). Of these, 306 of 359 (85.2%) of RCTs featured controlled treatment allocation patterns: where there was a consistent relationship between participants and treatment arms. The most frequent allocations patterns were ‘A + X & B + X’ (*i.e*. two treatments, each given unilaterally) and ‘A + A & B + B’ (*i.e*. two treatments, each given bilaterally or systemically). Uncontrolled treatment allocation was associated with unclear description in the RCT report (χ^2^ = 44.7, *p* < 0.001; Fig. 2A). Uncontrolled treatment allocation was also associated with two-eye patterns (Fisher’s exact test, *p* < 0.001), as just one of the 159 reported one-eye allocation patterns were uncontrolled (Fig. 2B). Three studies only featured a control arm with no treatment or placebo; one of these was uncontrolled as participants in the intervention group had one eye treated (‘A + X & X + X’). The other two studies were deemed ‘controlled’ in terms of allocation pattern, despite acknowledging an associated risk of performance bias affecting results through placebo effects [10].

**Table 2:** Treatment allocation patterns identified in 359 ophthalmological randomised-control trials published in 2022.

Allocation Pattern		Frequency
Controlled (*n* = 306)	A/B/C + A/B/C	1
	A/B + A/B	6
	A/B + A/B & C/B + C/B & D/B + D/B & E/B + E/B & F/B + F/B	1
	A + A & A/B + A/B & A/C + A/C	1
	A + A & B + B	82
	A + A & B + B & C + C	11
	A + A & B + B & C + C & D + D	4
	A + A & B + B & C + C & D + D & E + E	1
	A + A & B + B & C + C & D + D & E + E & F + F	1
	A + A & B + B & C + C & D + D & E + E & F + F & G + G	1
	A + A & B + B & X + X	1
	A + A & X + X	4
	A + B	31
	A + B & A + C	1
	A + B & C + B	2
	A + X	2
	A + X & B + X	140
	A + X & B + X & C + X	9
	A + X & B + X & C + X & D + X	4
	A + X & B + X & C + X & D + X & E + X	2
	A + X & B + X & X + X	1
Uncontrolled (*n* = 53)	A/B + X & A/B + A/B	1
	A + A & A + X & A + B & B + X & B + B	1
	A + A & B + B & A + B & A + X & B + X	1
	A + A & B + B & A + X & B + X	9
	A + A & B + B & A + X & B + X & A + B	5
	A + B & B + B	1
	A + X & A + A & B + X	1
	A + X & A + A & X + X	1
	A + X & B + B	2
	A + X & B + X & A + A & B + B	10
	A + X & B + X & A + A & B + B & A + B	5
	A + X & B + X & A + B	3
	A + X & B + X & C + X & A + A & B + B & C + C & A + B & A + C & B + C	2
	A + A & A + B	1
	A + A & B + B & C + C & A + X & B + X & C + X	2
	A + A & B + X	1
	A + X & A + A & B + X & B + B	3
	A + X & B + X & B + B & A + A	1
	A + X & B + X & C + X & D + X & A + A & B + B & C + C & D + D	1
	A + X & B + X & C + X & D + X & E + X & F + X & G + X & A + A & B + B & C + C & D + D & E + E & F + F & G + G	1
	A + X & X + X	1

In the allocation pattern notation, descending letters from ‘A’ describe treatments (including placebo), ‘X’ denotes no treatment, ‘+’ between letters corresponds to contralateral eyes within an archetypical individual, and ‘&’ separate archetypical individuals. Controlled allocation patterns were more common overall, and all but one patterns where one eye per participant was allocated were controlled.

### Uncontrolled treatment allocation is not associated with *p*-value noise or significance

A summary of the distribution of the characteristics of studies with controlled and uncontrolled treatment allocation is provided in Table 3. Some 711 *p*-values were extracted for primary outcomes from the ophthalmological RCTs: 611 from 306 RCTs with controlled treatment allocation, and 100 from 53 RCTs with uncontrolled treatment allocation. *p*-values tended to group around common accepted thresholds for statistical significance such as 0.05, 0.001, and 0.0001 (Fig. 2C), in-keeping with predictions based on widespread *p*-hacking or reporting bias [11, 12]. There was no significant difference in the recorded *p*-values in RCTs with controlled and uncontrolled treatment allocation (*t*-test, *t* = −0.603, *p* = 0.547); and the distribution of *p*-values in controlled and uncontrolled trials were very similar in terms of range, interquartile range, and median (Fig. 2D). Moreover, ‘significant’ results were not more common in controlled or uncontrolled trials, with thresholds of *p* < 0.05 (χ^2^ = 0.761, *p* = 0.383), *p* < 0.01 (χ^2^ = 0.052, *p* = 0.820), or *p* < 0.001 (χ^2^ < 0.001, *p* = 1.000).

**Table 3:** Characteristics of ophthalmological randomised control trials published between 1 January 2022 and 31 December 2022, stratified by whether their treatment allocation pattern was controlled or uncontrolled.

Study characteristic	n(controlled)	n(uncontrolled)	*p*-value
One-eye design	158	1	<0.001
Two-eye design	148	52	
Explicit allocation	150	10	<0.001
Implicit allocation	116	16	
Unclear allocation	40	27	
*p* < 0.05	295	43	0.383
*p* ≥ 0.05	316	57	
*p* < 0.01	200	31	0.820
*p* ≥ 0.01	411	69	
*p* < 0.001	43	7	1.000
*p* ≥ 0.001	568	93	
Academic funding	38	5	0.035
Academic and governmental funding	26	2	
Academic and industrial funding	7	0	
Academic, governmental, and industrial funding	4	0	
Governmental funding	28	6	
Governmental and industrial funding	4	0	
Industrial funding	80	9	
No funding / unclear	119	31	
Central America	1	0	0.098
East Asia	77	12	
Europe	55	12	
Middle East and North Africa	42	12	
Multiple / unclear	22	3	
North America	55	2	
Oceania	5	1	
South America	5	2	
South Asia	44	9	
Anglosphere countries	94	8	0.030
Non-Anglosphere countries	212	45	
Cataract	42	5	0.098
Cornea	105	11	
Glaucoma	30	7	
Neuro-ophthalmology	3	1	
Oculoplastics	15	7	
Paediatric ophthalmology	42	6	
Retina	62	14	
Strabismus	6	1	
Uveitis	1	1	
Medical intervention	230	32	0.038
Surgical intervention	76	21	

*p*-values for chi-squared tests (default) or Fisher’s exact test (where cells with fewer than five occurrences were present) are provided in the final column.

### Uncontrolled treatment allocation varies with funding source and is associated with non-English-speaking study settings and surgical interventions

The relative frequency of uncontrolled treatment allocation was associated with funding source (χ^2^ = 16.569, *p* = 0.035), but was not specifically associated with funding from industrial (χ^2^ = 2.115, *p* = 0.146), governmental (χ^2^ = 0.076, *p* = 0.783), or academic (χ^2^ = 1.514, *p* = 0.219) sources. Observed association was greatest with respect to industrial funding, but this did not meet the pre-specified criteria for statistical significance. There was also no association between uncontrolled treatment allocation and support from any funding or none (χ^2^ = 2.733, *p* = 0.098). This constellation of overall association without any specific relationships identified may have been due to variation being general rather than driven by a single type of funding, or because of smaller pairwise comparisons lacking sufficient statistical power to capture a relationship.

When association between controlled and uncontrolled treatment allocation was tested with respect to region of the world in which studies were conducted, the result indicated no specific association (Fisher’s exact test, *p* = 0.098). However, uncontrolled treatment allocation was over twice as frequent in studies conducted outside the anglosphere, and this association was statistically significant (χ^2^ = 4.681, *p* = 0.030).

Finally, uncontrolled treatment allocation was not associated with subspecialty of study (Fisher’s exact test, *p* = 0.098) but was more common in trials involving a surgical intervention than a medical intervention (χ^2^ = 4.287, *p* = 0.038). In surgical trials, 21 of 97 allocation patterns were uncontrolled; whereas in medical trials, only 32 of 262 allocation patterns were uncontrolled.

## Discussion

In this study, uncontrolled treatment allocation featured in a remarkable proportion of ocular randomised control trials: 14.5%. Despite peaks in the frequency of *p-*values around common levels of statistical significance—likely primarily due to reporting bias [12] rather than *p*-hacking [11] as many trials specified analytical plans in prospectively registered protocols—no evidence was found of uncontrolled treatment allocation leading to a greater chance of obtaining a statistically significant result, or of a systematic exploitation of uncontrolled treatment allocation by particular funders or world regions. Uncontrolled allocation was also more frequent in studies carried out in non-English-speaking countries, and where surgical interventions featured.

To describe treatment allocation in eye-related RCTs, Lee et al. grouped study designs as ‘one-eye’ or ‘two-eye’ based on whether one or both eyes of each study participant were included; and further split ‘two-eye’ designs based on whether treatments were different, the same, or random between eyes of each participant [2]. While one-eye design (*e.g*. ‘A + X & B + X’) is most common, significant heterogeneity in designs has been identified previously [2, 8]. However, the frequency of uncontrolled treatment allocation—where trials may be compromised *ipso facto* regardless of the analytical schema—has not previously been characterised. This study indicates that uncontrolled treatment allocation patterns are concerningly frequent, although not present in a majority of trials. As there is no evidence of this being an intentional process, the underlying reason is likely a systemic lack of education and emphasis of treatment allocation as an important aspect of trial design in ophthalmology. Notably, all but a single one-eye allocation pattern identified in this study were controlled. Therefore, promoting inclusion of one eye per participant for treatment and analysis could be a simpler means of promoting controlled treatment allocation. This may not be possible where the intervention or standard of care necessarily involves both eyes, but an emphasis on controlled allocation patterns may reduce the likelihood of uncontrolled treatment allocation.

In terms of limitations, this study did not characterise the relation of allocated interventions to standard of care in ophthalmology, or the quality of placebo controls. There is therefore further scope for investigation to explore the prevalence of suboptimal treatment allocation in RCTs, using well understood best practices such as control arms representing the established standard of care, or placebos minimising performance bias and maintaining participants’ blinding [6, 13]. These are important potential deficiencies because placebo effects are well documented in subjective and measured eye-related outcomes [14, 15]. Second, misclassification of studies with implicit or unclear reporting may have occurred, and further study could engage investigators or published RCT protocols to elucidate allocation patterns with greater confidence. Unclear description could be in part due to language barriers, which could explain part of the association between uncontrolled allocation patterns and non-anglosphere countries of study [16]. Third, the study was restricted to a single year; a sizable sample which permitted statistical analysis with sufficient power to draw meaningful conclusions, but a lack of ability to understand if the issues of uncontrolled and confounded RCTs is becoming more or less frequent over time. Work is ongoing to apply automated analysis of RCT reports and protocols to derive treatment allocation patterns, using large language models with ophthalmology-specific knowledge and reasoning ability [17, 18].

In view of the frequency of uncontrolled and unclear treatment allocation patterns, we would advocate initiatives to improve awareness, emphasis, and transparency of treatment allocation patterns in ophthalmological randomised control trials [2]. Subgroup analysis may be undertaken to describe the specific effect (or lack thereof) of uncontrolled treatment allocation, where present [19]. Controlled allocation patterns may be encouraged by including a mandatory treatment allocation parameter on clinical trial protocol registries, inclusion of treatment allocation pattern in ophthalmological extensions to consensus statements summarising best practices in clinical trials (*e.g*. CONSORT, SPIRIT), or addition of treatment allocation to critical appraisal tools for ophthalmological systematic reviews [20–22]. Promoting explicit inclusion of just one eye per participant is an alternative means of maximising the likelihood of treatment allocation being controlled; but may not always be feasible.

## Conclusion

Ophthalmological RCTs are frequently unclear in their reporting of treatment allocation, and uncontrolled treatment allocation patterns are concerningly frequent; with greater prevalence in surgical trials. Where treatment allocation patterns are reported explicitly or implicitly, the frequency of uncontrolled allocation is significantly lower. Initiatives to promote definition, reporting, and appraisal of treatment allocation patterns may thereby improve the statistical validity and evidence-quality of RCTs. This may require adoption of treatment allocation as a parameter within clinical trial registries, consensus statements, and critical appraisal tools.

## Summary

### What was known before

- Randomised-control trials (RCTs) are the bedrock of evidence-based ophthalmology, as they inform the community about which treatments lead to the best outcomes for patients In ophthalmology, RCTs are complicated by most participants having two eyes which may interact and affect study outcomes Treatment allocation may be uncontrolled, where the relationship between interventions and participants’ eyes is inconsistent.

### What this study adds

- Uncontrolled treatment allocation is a frequent issue in ophthalmological RCTs, identified in 14.8% of published studies Uncontrolled allocation is associated with two-eye allocation, surgical trials, and non-English-speaking countries A simple nomenclature system can be used to facilitate reporting treatment allocation patterns in study protocols, RCT reports, and in critical appraisal work (*e.g.* systematic review).

## Supplementary information

Supplementary Material 2

Supplementary Material 1

## Data Availability

All data required to replicate the study analysis are available as supplementary material. This provides researchers with a representative sample of ophthalmological randomised-control trials published over a 12-month period, for subsequent meta-research and analyses.

## References

[1] Armstrong RA. Statistical guidelines for the analysis of data obtained from one or both eyes. Ophthalmic Physiol Opt. 2013;33:7–14. 23252852 10.1111/opo.12009

[2] Lee CF, Cheng ACO, Fong DYT. Eyes or Subjects: Are Ophthalmic Randomized Controlled Trials Properly Designed and Analyzed?. Ophthalmology. 2012;119:869–72. 22226885 10.1016/j.ophtha.2011.09.025

[3] Raji S, Thirunavukarasu AJ, Taylor LJ, MacLaren RE. Functional vision tests as clinical trial outcome measures in ophthalmology: a scoping review. BMJ Open. 2025;15:e097970. 40436455 10.1136/bmjopen-2024-097970 PMC12121612

[4] Thabane A, Phillips MR, Wong TY, Thabane L, Bhandari M, Chaudhary V. The clinician’s guide to randomized trials: interpretation. Eye. 2022;36:481–2. 35058599 10.1038/s41433-021-01866-7 PMC8873262

[5] Piltz J, Gross R, Shin DH, Beiser JA, Dorr DA, Kass MA, Gordon MO. Contralateral effect of topical beta-adrenergic antagonists in initial one-eyed trials in the ocular hypertension treatment study. Am J Ophthalmol. 2000;130:441–53. 11024416 10.1016/s0002-9394(00)00527-4

[6] Maguire MG. Best practices for the design of clinical trials related to the visual system. Annu Rev Vis Sci. 2021;7:867–86. 34297597 10.1146/annurev-vision-093019-113930

[7] Prasad V, Gall V, Cifu A. The Frequency of Medical Reversal. Arch Intern Med. 2011;171:1675–6. 21747003 10.1001/archinternmed.2011.295

[8] Dong R, Ying G. Characteristics of design and analysis of ophthalmic randomized controlled trials. Ophthalmol Sci. 2023;3:100266. 36798523 10.1016/j.xops.2022.100266 PMC9926296

[9] Dickersin K, Hawkins BS, Le JT, et al. Building a reliable evidence base in eyes and vision. Br J Ophthalmol 2017; published online Oct 20.

[10] Margo CE. The placebo effect. Survey Ophthalmol. 1999;44:31–44. 10.1016/s0039-6257(99)00060-0 10466586

[11] Adda J, Decker C, Ottaviani M. P-hacking in clinical trials and how incentives shape the distribution of results across phases. Proc Natl Acad Sci. 2020;117:13386–92. 32487730 10.1073/pnas.1919906117 PMC7306753

[12] Easterbrook PJ, Berlin JA, Gopalan R, Matthews DR. Publication bias in clinical research. Lancet. 1991;337:867–72. 1672966 10.1016/0140-6736(91)90201-y

[13] Prasad VK, Cifu AS. Ending medical reversal: improving outcomes, Saving Lives. JHU Press, 2015.

[14] Imanaka T, Sato I, Tanaka S, Kawakami K. Predictive factors for the placebo effect in clinical trials for dry eye: a pooled analysis of three clinical trials. Br J Ophthalmol. 2017;101:1471–4. 28315833 10.1136/bjophthalmol-2016-309887

[15] Choe S, Kim YK, Chung W, Ko D, Lee M, Shim SR, Ha A. Placebo Effect and Its Determinants in Ocular Hypotensive Therapy: Meta-analysis and Multiple Meta-regression Analysis. Ophthalmology. 2023;130:1149–61. 37343706 10.1016/j.ophtha.2023.06.012

[16] Overcoming the language barrier in science communication. Nat Rev Bioeng 2023; 1: 305–305.

[17] Thirunavukarasu AJ, Mahmood S, Malem A, Foster WP, Sanghera R, Hassan R, et al. Large language models approach expert-level clinical knowledge and reasoning in ophthalmology: A head-to-head cross-sectional study. PLoS Digital Health. 2024;3:e0000341. 38630683 10.1371/journal.pdig.0000341 PMC11023493

[18] Sanghera R, Thirunavukarasu AJ, El Khoury M, et al. High-performance automated abstract screening with large language model ensembles. JAMIA 2025; ocaf050. 10.1093/jamia/ocaf050 PMC12012331 40119675

[19] Kent DM, Rothwell PM, Ioannidis JP, Altman DG, Hayward RA. Assessing and reporting heterogeneity in treatment effects in clinical trials: a proposal. Trials. 2010;11:85. 20704705 10.1186/1745-6215-11-85 PMC2928211

[20] Chan A-W, Tetzlaff JM, Gøtzsche PC, Altman DG, Mann H, Berlin JA, et al. SPIRIT 2013 explanation and elaboration: guidance for protocols of clinical trials. BMJ. 2013;346:e7586. 23303884 10.1136/bmj.e7586 PMC3541470

[21] Schulz KF, Altman DG, Moher D, CONSORT Group. CONSORT 2010 statement: updated guidelines for reporting parallel group randomised trials. BMJ. 2010;340:c332. 20332509 10.1136/bmj.c332 PMC2844940

[22] Joksimovic L, Koucheki R, Popovic M, Ahmed Y, Schlenker MB, Ahmed IIK. Risk of bias assessment of randomised controlled trials in high-impact ophthalmology journals and general medical journals: a systematic review. Br J Ophthalmol. 2017;101:1309–14. 28659390 10.1136/bjophthalmol-2017-310313

