# Supplementary material for: Treatment allocation in ophthalmological randomised-control trials (TAO-RCT): A cross-sectional meta-research study
Source: Eye (Lond). 2025 Jul 17;39(13):2591–8. doi: 10.1038/s41433-025-03922-y (PMC12402246; doi:10.1038/s41433-025-03922-y)
Supplement: Supplementary file 2 — Supplementary Material 1 [file 41433_2025_3922_MOESM2_ESM.pdf]

Supplementary Material 1: Literature search undertaken for the systematic review

The Cochrane Library

	Search Terms	N
**#1**	[mh Ophthalmology] OR [mh “Ophthalmologic Surgical Procedures”] OR [mh “Eye Disease”] OR [mh Eye] OR [mh “Eye Surgery”] OR [mh “Visual Disorder”] OR (eye OR ophthalmolog*):ti,ab,kw	53,911
**#2**	[mh “Glaucoma, Angle-Closure”] OR [mh “Glaucoma, Open-Angle”] OR [mh Glaucoma] OR [mh “Glaucoma, Neovascular”] OR [mh “Glaucoma Drainage Implants”] OR [mh “Low Tension Glaucoma”] OR glaucom*:ti,ab,kw	8,909
**#3**	[mh Retina] OR [mh “Retinal Diseases”] OR [mh “Diabetic Retinopathy”] OR [mh “Epiretinal Membrane”] OR [mh “Retinal Artery Occlusion”] OR [mh “Retinal Degeneration”] OR [mh “Macular Degeneration”] OR [mh “Retinal Detachment”] OR [mh “Retinal Haemorrhage”] OR [mh “Retinal Neovascularization”] OR [mh “Retinal Vasculitis”] OR [mh “Retinal Drusen”] OR [mh “Retinal Vein Occlusion”] OR [mh Retinitis] OR [mh Chorioretinitis] OR [mh “Retinopathy of Prematurity”] OR [mh “Vitreoretinopathy, Proliferative”] OR [mh “Vitreous Detachment”] OR (retina* OR retinopath* OR vitreoretinopath* OR epiretinal membrane* OR macular degenerat* OR maculopath* OR (macula* adj (edema* or oedema*)) OR retinitis OR chorioretinitis OR retinopathy OR vitreoretinal):ti,ab,kw	18,873
**#4**	[mh Cornea] OR [mh “Corneal Diseases”] OR [mh “Corneal Opacity”] OR cornea*:ti,ab,kw	11,351
**#5**	[mh “Cataract Extraction”] OR [mh Cataract] OR (cataract OR phacoemulsification):ti,ab,kw	9,495
**#6**	[mh “Scleral Diseases”] OR [mh “Uveal Diseases”] OR [mh “Choroid Diseases”] OR [mh “Eyelid Diseases”] OR [mh “Iris Diseases”] OR [mh Uveitis] OR [mh Panuveitis] OR [mh “Uveitis, Anterior”] OR [mh “Uveitis, Posterior”] OR [mh “Uveitis, Intermediate”] OR (iridocyclitis OR iritis OR uveitis OR panuveitis):ti,ab,kw	3,446
**#7**	[mh Strabismus] OR [mh Amblyopia] OR (strabismus OR amblyopia):ti,ab,kw	2,042
**#8**	[mh “Refractive Errors”] OR [mh Myopia] OR [mh Hyperopia] OR [mh Presbyopia] OR [mh Astigmatism] OR (refractive error OR myopia OR hyperopia OR presbyopia OR ametropia OR astigmatism):ti,ab,kw	6,219
**#9**	[mh Blepharitis] OR [mh Chalazion] OR [mh Ectropion] OR [mh Entropion] OR [mh Hordeolum] OR (blepharitis OR chalazion OR ectropion OR entropion OR hordeolum):ti,ab,kw	643
**#10**	[mh “Eye Neoplasms”] OR [mh “Conjunctival Neoplasms”] OR [mh “Eyelid Neoplasms”] OR [mh “Orbital Neoplasms”] OR [mh “Retinal Neoplasms”] OR [mh Retinoblastoma] OR [mh “Uveal Neoplasms”] OR [mh “Choroid Neoplasms”] OR [mh “Iris Neoplasms”] OR (retinoblastoma* OR (conjunctival adj (neoplasm* OR tumo?r* OR malignan* OR cancer*)) OR (iris adj (neoplasm* OR tumo?r* OR malign* OR cancer*)) OR (orbital adj (neoplasm* OR tumo?r* OR malignan* OR cancer*))):ti,ab,kw	365
**#11**	#1 OR #2 OR #3 OR #4 OR #5 OR #6 OR #7 OR #8 OR #9 OR #10	67,660
**#12**	[mh “Randomized Controlled Trial”] OR [mh “Controlled Clinical Trial”] OR [mh “Clinical Trial, Phase III”] OR [mh “Clinical Trial, Phase IV”]:ti,ab,kw	38,477
**#13**	(“randomi*sed control* trial” OR “phase iii” OR “phase 3” OR “phase iv” OR “phase 4”):ti,ab,kw	82,826
**#14**	#12 OR #13	118,391
**#15**	#11 AND #14	3,921
**#16**	#15 AND year=2022	421

Embase

	Search Terms	N
**#1**	exp Ophthalmology/ OR exp Ophthalmologic Surgical Procedures/ OR exp Eye Disease/ OR exp Eye/ OR exp Eye Surgery/ OR exp Visual Disorder/	1,351,104
**#2**	exp Glaucoma, Angle-Closure/ OR exp Glaucoma, Open-Angle/ OR exp Glaucoma/ OR exp Glaucoma, Neovascular/ OR exp Glaucoma Drainage Implants/ OR exp Low Tension Glaucoma/ OR glaucom*.mp.	116,368
**#3**	exp Retina/ OR exp Retinal Diseases/ OR exp Diabetic Retinopathy/ OR exp Epiretinal Membrane/ OR exp Retinal Artery Occlusion/ OR exp Retinal Degeneration/ OR exp Macular Degeneration/ OR exp Retinal Detachment/ OR exp Retinal Haemorrhage/ OR exp Retinal Neovascularization/ OR exp Retinal Vasculitis/ OR exp Retinal Drusen/ OR exp	519,870

	Retinal Vein Occlusion/ OR exp Retinitis/ OR exp Chorioretinitis/ OR exp Retinopathy of Prematurity/ OR exp Vitreoretinopathy, Proliferative/ OR exp Vitreous Detachment/ OR (retina* OR retinopath* OR vitreoretinopath* OR epiretinal membrane* OR macular degenerat* OR maculopath* OR (macula* adj (edema* or oedema*)) OR retinitis OR chorioretinitis OR retinopathy OR vitreoretinal).mp.	
**#4**	exp Cornea/ OR exp Corneal Diseases/ OR exp Corneal Opacity/ OR cornea*.mp.	188,114
**#5**	exp Cataract Extraction/ OR exp Cataract/ OR (cataract OR phacoemulsification).mp.	113,195
**#6**	exp Scleral Diseases/ OR exp Uveal Diseases/ OR exp Choroid Diseases/ OR exp Eyelid Diseases/ OR exp Iris Diseases/ OR exp Uveitis/ OR exp Panuveitis/ OR exp Uveitis, Anterior/ OR exp Uveitis, Posterior/ OR exp Uveitis, Intermediate/ OR (iridocyclitis OR iritis OR uveitis OR panuveitis).mp.	160,224
**#7**	exp Strabismus/ OR strabismus.mp. OR exp Amblyopia/ OR amblyopia.mp.	41,143
**#8**	exp Refractive Errors/ OR exp Myopia/ OR exp hyperopia/ OR exp Presbyopia/ OR exp Astigmatism/ OR (refractive error OR myopia OR hyperopia OR presbyopia OR ametropia OR astigmatism).mp.	70,993
**#9**	exp Blepharitis/ OR exp Chalazion/ OR exp Ectropion/ OR exp Entropion/ OR exp Hordeolum/ OR (blepharitis OR chalazion OR ectropion OR entropion OR hordeolum).mp.	11,249
**#10**	exp Eye Neoplasms/ OR exp Conjunctival Neoplasms/ OR exp Eyelid Neoplasms/ OR exp Orbital Neoplasms/ OR exp Retinal Neoplasms/ OR exp Retinoblastoma/ OR exp Uveal Neoplasms/ OR exp Choroid Neoplasms/ OR exp Iris Neoplasms/ OR (retinoblastoma* OR (conjunctival adj (neoplasm* OR tumo?r* OR malignan* OR cancer*)) OR (iris adj (neoplasm* OR tumo?r* OR malign* OR cancer*)) OR (orbital adj (neoplasm* OR tumo?r* OR malignan* OR cancer*))).mp.	84,825
**#11**	#1 OR #2 OR #3 OR #4 OR #5 OR #6 OR #7 OR #8 OR #9 OR #10	1,452,403
**#12**	exp Randomized Controlled Trial/ OR exp Controlled Clinical Trial/ OR exp Clinical Trial, Phase III/ OR exp Clinical Trial, Phase IV/	983,824
**#13**	(“randomi*sed control* trial” OR “phase iii” OR “phase 3” OR “phase iv” OR “phase 4”).mp.	234,319
**#14**	#12 OR #13	1,129,111
**#15**	#11 AND #14	48,366
**#16**	#15 AND year=2022	2573

MEDLINE

	**Search Terms**	**N**
**#1**	exp Ophthalmology/ OR exp Ophthalmologic Surgical Procedures/ OR exp Eye Disease/ OR exp Eye/ OR exp Eye Surgery/ OR exp Visual Disorder/	856,080
**#2**	exp Glaucoma, Angle-Closure/ OR exp Glaucoma, Open-Angle/ OR exp Glaucoma/ OR exp Glaucoma, Neovascular/ OR exp Glaucoma Drainage Implants/ OR exp Low Tension Glaucoma/ OR glaucom*.mp.	83,231
**#3**	exp Retina/ OR exp Retinal Diseases/ OR exp Diabetic Retinopathy/ OR exp Epiretinal Membrane/ OR exp Retinal Artery Occlusion/ OR exp Retinal Degeneration/ OR exp Macular Degeneration/ OR exp Retinal Detachment/ OR exp Retinal Haemorrhage/ OR exp Retinal Neovascularization/ OR exp Retinal Vasculitis/ OR exp Retinal Drusen/ OR exp Retinal Vein Occlusion/ OR exp Retinitis/ OR exp Chorioretinitis/ OR exp Retinopathy of Prematurity/ OR exp Vitreoretinopathy, Proliferative/ OR exp Vitreous Detachment/ OR (retina* OR retinopath* OR vitreoretinopath* OR epiretinal membrane* OR macular degenerat* OR maculopath* OR (macula* adj (edema* or oedema*)) OR retinitis OR chorioretinitis OR retinopathy OR vitreoretinal).mp.	361,996
**#4**	exp Cornea/ OR exp Corneal Diseases/ OR exp Corneal Opacity/ OR cornea*.mp.	145,977
**#5**	exp Cataract Extraction/ OR exp Cataract/ OR (cataract OR phacoemulsification).mp.	79,018
**#6**	exp Scleral Diseases/ OR exp Uveal Diseases/ OR exp Choroid Diseases/ OR exp Eyelid Diseases/ OR exp Iris Diseases/ OR exp Uveitis/ OR exp Panuveitis/ OR exp Uveitis, Anterior/ OR exp Uveitis, Posterior/ OR exp Uveitis, Intermediate/ OR (iridocyclitis OR iritis OR uveitis OR panuveitis).mp.	96,010
**#7**	exp Strabismus/ OR strabismus.mp. OR exp Amblyopia/ OR amblyopia.mp.	29,070
**#8**	exp Refractive Errors/ OR exp Myopia/ OR exp hyperopia/ OR exp Presbyopia/ OR exp Astigmatism/ OR (“refractive error” OR “myopia” OR “hyperopia” OR “presbyopia” OR “ametropia” OR “astigmatism”).mp.	51,920

**#9**	exp Blepharitis/ OR exp Chalazion/ OR exp Ectropion/ OR exp Entropion/ OR exp Hordeolum/ OR (blepharitis OR chalazion OR ectropion OR entropion OR hordeolum).mp.	6,777
**#10**	exp Eye Neoplasms/ OR exp Conjunctival Neoplasms/ OR exp Eyelid Neoplasms/ OR exp Orbital Neoplasms/ OR exp Retinal Neoplasms/ OR exp Retinoblastoma/ OR exp Uveal Neoplasms/ OR exp Choroid Neoplasms/ OR exp Iris Neoplasms/ OR (retinoblastoma* OR (conjunctival adj (neoplasm* OR tumo?r* OR malignan* OR cancer*)) OR (iris adj (neoplasm* OR tumo?r* OR malign* OR cancer*)) OR (orbital adj (neoplasm* OR tumo?r* OR malignan* OR cancer*))).mp.	61,111
**#11**	#1 OR #2 OR #3 OR #4 OR #5 OR #6 OR #7 OR #8 OR #9 OR #10	988,420
**#12**	exp Randomized Controlled Trial/ OR exp Controlled Clinical Trial/ OR exp Clinical Trial, Phase III/ OR exp Clinical Trial, Phase IV/	698,009
**#13**	(“randomi*sed control* trial” OR “phase iii” OR “phase 3” OR “phase iv” OR “phase 4”).mp.	112,437
**#14**	#12 OR #13	761,396
**#15**	#11 AND #14	22,163
**#16**	#15 AND year=2022	693
